# ASSESSING THE PREDICTIVE VALUE OF THE C-REACTIVE PROTEIN AND NEUTROPHIL-TO-LYMPHOCYTE RATIO COMBINED SCORE FOR ORGAN FAILURE IN ACUTE PANCREATITIS

**DOI:** 10.1590/S0004-2803.24612024-119

**Published:** 2025-06-16

**Authors:** Jean Félix PIÑERÚA-GONSÁLVEZ, María Lourdes RUIZ-REBOLLO, Luis FERNÁNDEZ-SALAZAR

**Affiliations:** 1 Department of Gastroenterology, Hospital Clinico Universitario de Valladolid, Valladolid, Spain.

**Keywords:** Pancreatitis, multiple organ failure, C-reactive protein, organ dysfunction scores, Pancreatite, falência de múltiplos órgãos, proteína C-reativa, escores de disfunção orgânica

## Abstract

**Background:**

Acute pancreatitis (AP) is a common gastrointestinal disorder, with organ failure being the primary cause of mortality. This study evaluates the combined score of C-reactive protein level and neutrophil-to-lymphocyte ratio (C-NLR score), as a predictor of organ failure.

**Methods:**

A retrospective analysis was conducted on AP patients admitted to the Hospital Clínico Universitario de Valladolid between March 2014 and January 2022. The C-NLR score was calculated from admission data: patients with both elevated neutrophil-to-lymphocyte ratio (NLR) and C-reactive protein (CRP) were assigned a score of 2, those with one elevated marker received a score of 1, and a score of 0 was given to those with neither.

**Results:**

A total of 778 patients were included: 611 with mild AP (MAP), 123 with moderately severe AP (MSAP), and 44 with severe AP (SAP). A C-NLR score of 2 was most frequently observed in patients with MSAP (56.1%) and SAP (56.85%), while a score of 1 was more common in patients with MAP (46.9%). Multivariable logistic regression analysis revealed that a C-NLR score of 2 significantly increased the likelihood of organ failure by threefold (OR 3.176; 95% CI 1.297-7.775).

**Conclusion:**

The C-NLR score could be a useful supplementary tool for predicting organ failure in AP, complementing traditional scoring systems.

## RESUMO

**Contexto:**

A pancreatite aguda (PA) é um distúrbio gastrointestinal comum, sendo a falência de órgãos a principal causa de mortalidade. Este estudo avalia o escore combinado do nível de proteína C reativa e da relação neutrófilo-linfócito (escore C-NLR), como preditor de falência de órgãos.

**Métodos:**

Foi realizada uma análise retrospectiva de pacientes com PA internados no Hospital Clínico Universitário de Valladolid entre março de 2014 e janeiro de 2022. O escore C-NLR foi calculado a partir dos dados de admissão: pacientes com relação neutrófilo-linfócito (NLR) e proteína C reativa (PCR) elevadas receberam uma pontuação de 2, aqueles com um marcador elevado receberam uma pontuação de 1 e uma pontuação de 0 foi dada àqueles sem nenhum dos dois.

**Resultados:**

Um total de 778 pacientes foram incluídos: 611 com PA leve (PAM), 123 com PA moderadamente grave (PASM) e 44 com PA grave (PAS). Um escore C-NLR de 2 foi mais frequentemente observado em pacientes com PASM (56,1%) e PAS (56,85%), enquanto um escore de 1 foi mais comum em pacientes com PAM (46,9%). A análise de regressão logística multivariada revelou que uma pontuação C-NLR de 2 aumentou significativamente a probabilidade de falência de órgãos em três vezes (OR 3,176; IC 95% 1,297-7,775).

**Conclusão:**

O escore C-NLR pode ser uma ferramenta complementar útil para predizer falência de órgãos na PA, complementando os sistemas de pontuação tradicionais.

## INTRODUCTION

Acute pancreatitis (AP) is a common inflammatory gastrointestinal disorder worldwide. Over the past two decades, there has been a rise in the incidence and hospitalization rates associated with AP. In the United States alone, approximately 275,000 admissions occur annually due to this condition, placing a significant economic burden on healthcare systems with hospitalization costs surpassing $30,000 per individual^1^ ^,^ ^2^. Most of the patients are admitted with a mild form of the disease and have brief hospital stays. However, in approximately 20% of cases where the disease manifests severely, the mortality rate increases to 30-50%^3^. This notable increase in mortality is mainly a result of its association with multiple organ failure and systemic inflammatory response syndrome (SIRS)^4^ ^,^ ^5^. 

Several comprehensive studies have shown the importance of the first 24 hours after symptom onset in identifying patients who are at risk of developing severe forms of AP^6^ ^-^ ^9^. Consequently, many biomarkers have been studied to assess AP severity within this critical time window. However, none of them have been recognized as definite instruments with universally applicable value or consistent accuracy^10^ ^,^ ^11^. Thus, efforts to develop accurate and easy-to-use predictive indicators continue, aiming to improve outcomes and reduce mortality rates.

C-reactive protein (CRP) is a pentraxin synthesized by the hepatocytes in response to inflammatory mediators such as interleukin 6 (IL-6), tumor necrosis factor α (TNFα) and interleukin 1 β (IL-1β). Elevated serum levels of this acute-phase protein are observed in response to various conditions including infections, inflammatory diseases, trauma, and cancer^12^. In the same way, the neutrophil-to-lymphocyte ratio (NLR) has been investigated as an indicator of inflammatory processes, stress responses, and a prognostic factor in various cancers and benign inflammatory conditions such as AP^13^.

The combined score of CRP level and NLR (C-NLR score) has been recently studied as a novel biomarker to predict clinical outcomes in some cancers, acute myocardial infarction, and infectious diseases^14^ ^-^ ^18^. This study is aimed to assess the value of C-NLR score to predict organ failure in AP.

## METHODS

### Study design

A retrospective analysis was conducted using a prospective database of patients diagnosed with AP, who were admitted to the Department of Gastroenterology at *Hospital Clínico Universitario de Valladolid*, Spain, between March 2014 and January 2022. Based on an estimated incidence of moderately severe acute pancreatitis and severe acute pancreatitis of 35%^19^, a confidence level of 95%, and a margin of error of 5%, the minimum sample size required for adequate statistical power was calculated to be 350 patients. Patients who were under 18 years of age, had a history of recurrent pancreatitis, previously diagnosed with chronic pancreatitis, undergone pancreatic surgery, had a history of cancer, were pregnant, referred from other hospitals, or had incomplete data in the electronic medical record were excluded from the study.

The study followed the guidelines of the reporting of studies conducted using Observational routinely-collected data (RECORD) and was approved by the ethics committee of *Hospital Clínico Universitario de Valladolid*, Spain under approval number PI-24-524-C.

### Data collection

Data were obtained from electronic medical records of *Hospital Clínico Universitario de Valladolid*. Information collected included patient clinical demographic data, etiology, severity (revised Atlanta criteria, Ranson criteria and Bedside Index of Severity in Acute Pancreatitis [BISAP]), laboratory findings at admission, length of stay, local complications, organ failure, systemic complications and mortality. 

### Study definitions and classifications

Based on the revision of the Atlanta classification, the diagnosis of AP required the presence of at least two of the following criteria: abdominal pain consistent with AP, biochemical evidence of pancreatitis (amylase or lipase levels elevated to at least three times the upper limit of normal), and/or identifiable characteristic findings observed through contrast-enhanced computed tomography (CECT), magnetic resonance imaging (MRI), or transabdominal ultrasonography^20^.

Pancreatitis severity was categorized into the following three groups: mild acute pancreatitis (MAP) indicated the absence of organ failure and both local or systemic complications, moderately severe acute pancreatitis (MSAP) denoted the presence of transient organ failure or local or systemic complications in the absence of persistent organ failure, and severe acute pancreatitis (SAP) referred to the presence of persistent organ failure involving one or more organs and lasting beyond 48 hours^20^. 

Organ failure is characterized by a score of ≥2 points for any of the three organs (respiratory, cardiovascular, or renal system) according to the modified Marshall scoring system^21^. On the other hand, systemic complications were defined as the worsening of pre-existing comorbidities, such as heart failure, coronary artery disease, chronic lung disease, or chronic kidney disease, triggered by AP. Whereas local complication refers to the presence of acute peripancreatic fluid collection, pancreatic pseudocyst, acute necrotic collection and walled-off necrosis^20^.

In this study, mortality was defined as any event resulting in the death of the patient during their hospital admission or within 90 days after their discharge.

The NLR was determined by dividing the absolute count of neutrophils by the absolute count of lymphocytes. A C-NLR score of 2 was assigned to patients identified as having both a high NLR and a high CRP level (>5 mg/L), a score of 1 was given to those with either a high NLR or a high CRP level, and a score of 0 was assigned to patients with neither a high NLR nor a high CRP level.

### Statistical analysis

In case of normal distribution, continuous variables were represented as the mean ± standard deviation (SD), whereas for non-normal distributions, the median and interquartile range were utilized. Categorical variables were expressed as frequencies and proportions. Chi-square or 2-tailed Fisher’s exact test were applied for categorical data. For continuous variables, the groups were compared by using one-way analysis of variance (ANOVA) with post-hoc correction or Kruskal-Wallis test according to the result normality test. The optimal NLR cutoff was established through the execution of a receiver operating characteristic (ROC) curve analysis and the calculation of the Youden index. The predictiveness of the C-NLR score for organ failure was assessed by calculating odds ratios (ORs) and their 95% confidence intervals (95% CIs) using both univariable and multivariable logistic regression analysis. In the multivariate analysis, only the variables that achieved a *P*-value <0.1 in the univariate analysis were included. Statistical analyzes were calculated by using IBM® SPSS® Statistics 21.0.

## RESULTS

During the study period, a total of 992 patients with AP were admitted to the gastroenterology department. Among them, 778 patients were included in the current study. Of these patients, 416 (53.5%) were female. The mean age was 68.6±16.9 years. Among the diverse etiologies of AP, biliary etiology was the most prevalent, accounting for 63.1% of cases. In terms of pre-existing chronic conditions, hypertension was reported in 53.9% of the patients, while diabetes mellitus was documented in 16.7% of the cases. Regarding lifestyle habits, 16.2% were smokers, and 23.5% drank alcohol. A comprehensive summary of baseline patient characteristics is provided in Table 1.

**TABLE 1:** Baseline characteristics.

Variables	n=778
Age (years), mean (± SD)	68.6 (±16.9)
Sex, n (%)	
Male	362 (46.5)
Female	416 (53.5)
BMI, (kg/m^2^) mean (±SD)	27.8 (±4.7)
WC (cm), mean (±SD)	100.4 (± 14.2)
Etiologies, n (%)	
Biliary	491 (63.1)
Alcohol	57 (7.3)
ERCP	28 (3.6)
Idiopathic	157 (20.2)
Others*	45 (5.8)
Diabetes mellitus, n (%)	130 (16.7)
Hypertension, n (%)	419 (53.9)
Alcoholism, n (%)	173 (22.2)
Smoker, n (%)	125 (16.1)

SD: standard deviation; BMI: body mass index; WC: waist circumference; ERCP: endoscopic retrograde cholangiopancreatography. *The “Others” category included less common etiologies of acute pancreatitis in the present cohort, including hypertriglyceridemia, drug-induced pancreatitis, autoimmune pancreatitis, and pancreas divisum.

According to the revised Atlanta Classification, 611 patients (78.5%) were categorized as MAP, while 123 patients (15.8%) were classified as MSAP and 44 patients (5.7%) as SAP. Compared to the MAP and MSAP groups, the SAP group had a significantly higher incidence of organ failure, systemic complications, longer hospital stays, and higher mortality rates (Table 2).

Both the MSAP and SAP groups had elevated white blood cell (WBC) counts and CRP levels compared to the MAP group. Additionally, the MSAP group had a higher absolute neutrophil count (ANC) and NLR than the MAP group, but no significant differences were found between the MSAP and SAP groups. Furthermore, there were no significant differences in absolute lymphocyte count (ALC) among the three groups (Table 2).

**TABLE 2:** Comparison of clinical outcomes and laboratorial data among severity categories in patients with acute pancreatitis.

Variables	MAP (n=611)	MSAP (n=123)	SAP (n=44)	***P* value** ^*&*^
Ranson Score, median (IQR)	1 (1-2)	2 (1-3) ^*a*^	2 (1-3) ^*a*^	<0.001
BISAP, median (IQR)	1 (0-1)	2 (1-3)^*a*^	2 (1-3) ^*ab*^	<0.001
Organ failure, n (%)	0 (0)	41 (33.3)^*a*^	43 (97.7)^*ab*^	<0.001
Local complications, n (%)	0 (0)	55 (44.7)^*a*^	17 (38.6)^*a*^	<0.001
Systemic complications, n (%)	0 (0)	58 (47.2^*a*^	29 (65.9)^*ab*^	<0.001
Length of stay (days), median (IQR)	5 (4-7)	13 (8-20)^*a*^	10 (3-25)^*ab*^	<0.001
Mortality, n (%)	0 (0)	0 (0)	29 (65.9)^*ab*^	<0.001
WBC (10^9^/L), median (IQR)	11.3 (8.9-14.5)	14.2 (10.9-18.5)^*a*^	13.6 (10.9-16.6)^*a*^	<0.001
ANC (10^9^/L), median (IQR)	9.2 (6.6-12.4)	12.3 (8.7-16.1)^*a*^	11.0 (7.5-14.3)	<0.001
ALC (10^9^/L), median (IQR)	1.2 (0.7-1.7)	0.9 (0.6-1.5)	0.9 (0.4-2.0)	0.078
NLR (IQR)	7.6 (4.2-15.0)	12.4 (7.3-19.4)^*a*^	11.3 (5.7-21.9)	<0.001
CRP (mg/L), median (IQR)	10.9 (3.7-38.2)	25.0 (6.0-92.0)^*a*^	47.1 (12.5-132.5)^*a*^	<0.001

IQR: interquartile range; BISAP: Bedside Index of Severity in Acute Pancreatitis; WBC: white blood count; ANC: absolute neutrophil count; ALC: absolute lymphocyte count; NLR: neutrophil-to-lymphocyte ratio; CRP: C-reactive protein; MAP: mild acute pancreatitis; MSAP: moderately severe acute pancreatitis; SAP: severe acute pancreatitis. ^&^Comparison among the three groups. The significance for pairwise comparisons was calculated using the Bonferroni adjustment. ^a^ *P*<0.05, compared with MAP; ^b^ *P*<0.05, compared with MSAP.

**FIGURE 1:**
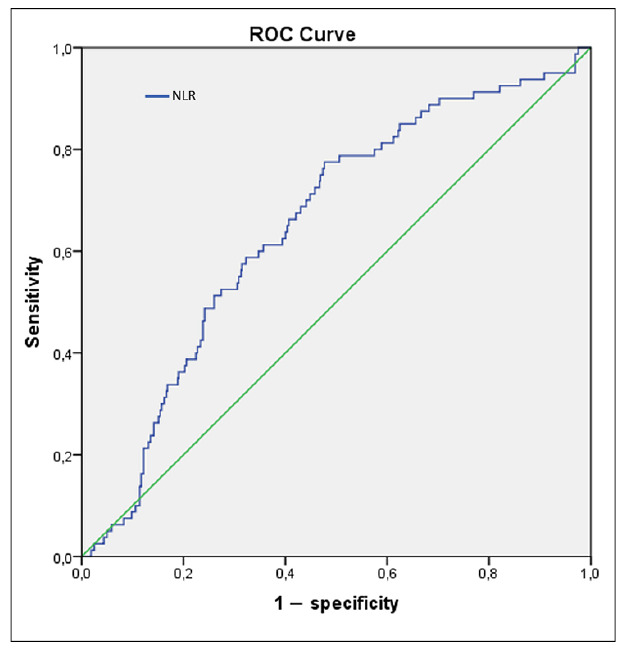
Receiver operating characteristic curve for NLR to predict organ failure in patients with acute pancreatitis.

ROC: Receiver-operating characteristic; NLR: neutrophil-to-lymphocyte ratio.

The area under the ROC curve (AUC) for NLR in predicting organ failure was found to be 0.65 (95%CI 0.59-0.71; *P*<0.001). Based on Youden’s index, the optimal cut-off point for NLR that differentiated patients with organ failure from those who did not develop organ failure was determined to be 8.41 (sensitivity 76.1%, specificity 52.0%) (Figure 1). This cut-off was used to classify patients with a high NLR in the C-NLR score. 

**FIGURE 2:**
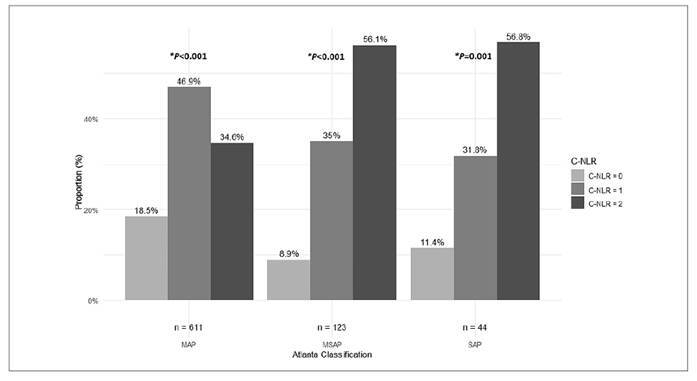
Distribution of C-NLR score among severity categories in acute pancreatitis patients.

MAP: mild acute pancreatitis; MSAP: moderately severe acute pancreatitis; SAP: severe acute pancreatitis; C-NLR: C-reactive protein and neutrophil-to-lymphocyte ratio combined score. *Significance was calculated using the chi-square test for homogeneity.

A C-NLR score of 2 was most frequently observed in patients with MSAP (56.1%; *P*<0.001) and SAP (56.85%; *P*=0.001), while patients with MAP exhibited a higher frequency of C-NLR scores of 1 (46.9%; *P*<0.001), as illustrated in Figure 2.

In the multivariable logistic regression analysis, a C-NLR score of 2 was identified as a significant predictor of organ failure, increasing the likelihood of its development by threefold (OR 3.176; 95%CI 1.297-7.775). Additionally, a C-NLR score of 1 was also assessed, demonstrating an odds ratio of 1.399 (95%CI 0.550-3.556), indicating a moderate association with the risk of organ failure (Table 3).

**TABLE 3:** Univariable and multivariable analysis to assess the relationship between C-NLR score and organ failure in patients with acute pancreatitis.

	Nonadjusted analysis		Adjusted analysis	
Variables	OR (95%CI)	***P* value**	OR (95%CI)	***P* value**
Sex				
Male	0.995 (0.632-1.567)	0.984		
Female	1.005 (0.638-1.582)	0.984		
Age	1.037 (1.020-1.055)	<0.001	1.021 (1.001-1.040)	0.038
Hypertension	3.829 (2.203-6.655)	<0.001	2.099 (1.138-3.873)	0.018
Diabetes mellitus	2.718 (1.643-4.497)	<0.001	1.968 (1.153-3.357)	0.013
BMI	1,052 (1.005-1.101)	0.028	1.046 (0.994-1.101)	0.083
C-NLR				
1	2.127 (1.295-3.494)	0.003	1.399 (0.550-3.556)	0.481
2	3.177 (1.981-5.095)	<0.001	3.176 (1.297-7.775)	0.011

OR: odds ratio; 95%CI: 95% confidence interval; BMI: body mass index; C-NLR: C-reactive protein and neutrophil-to-lymphocyte ratio combined score.

## DISCUSSION

Scoring systems have been developed to identify and treat high-risk patients with AP, aiming to improve outcomes and reduce mortality rates. However, these current scores have several limitations. For instance, the Ranson criteria require a 48-hour waiting period for an accurate assessment, the BISAP score has low positive predictive value and sensitivity for predicting mortality, and the APACHE II score is complex due to the numerous parameters it considers. Consequently, much research has been conducted to develop new prognostic markers that are simpler, faster, and more cost-effective^22^ ^-^ ^24^.

CRP is a pentraxin produced by the liver mainly in response to inflammatory mediators such as interleukin 6 (IL-6), tumor necrosis factor α (TNFα), and interleukin 1 β (IL-1β). Following an inflammatory stimulus, CRP secretion begins within 4 to 6 hours, steadily doubling every 8 hours and reaching a peak between 36 and 50 hours afterward. Once the stimulus disappears, CRP levels drop rapidly, given its half-life of 19 hours. The levels of this acute-phase protein in the serum increase due to infections, inflammatory diseases, trauma, and cancer^12^. Several studies have identified this acute-phase protein as a reliable outcome predictor in critically ill patients^12^ ^,^ ^25^. Particularly in the context of AP, CRP has demonstrated accurate and discriminative value for predicting SAP, pancreatic necrosis, and in-hospital mortality^12^.

Regarding neutrophil-to-lymphocyte ratio (NLR), initially described by Zahorec et al.^26^, provides insight into the immune response by accounting for two opposing and complementary components of the immune pathway. Neutrophils, as the central cell type in the innate immune system, are closely associated with inflammatory activation, while lymphocytes, the primary cells of the adaptive immune system, play a key role in regulating inflammation. As a result, NLR reflects the balance between inflammatory activation and its regulation. Consequently, a higher NLR value signifies a more imbalanced inflammatory state^27^ ^,^ ^28^. An increased mortality risk in infections, malignant tumors, and cardiovascular diseases has been correlated to elevated NLR^29^. The prognostic value of this inflammatory biomarker in AP has been the subject of research over the past decade. For instance, Azab *et al*. discovered that, in patients with AP, an NLR greater than 4.7 predicted ICU admission and prolonged hospitalization more accurately than total leukocyte count or the absolute counts of neutrophils and lymphocytes individually^30^. In another study, Suppiah et al. found that elevated NLR was significantly associated with SAP. However, in contrast to Azab et al., Suppiah’s study identified different optimal NLR cut-offs at various time points: 10.6 on day 0, 8.1 on day 1, and 4.8 on day 2 of admission^31^.

The C-NLR score is a novel inflammatory marker that has been studied in recent years as a prognostic indicator for various clinical outcomes, including gastric cancer, non-small-cell lung cancer, and acute myocardial infarction in patients undergoing percutaneous coronary intervention, as well as COVID-19 pneumonia^14^ ^-^ ^17^. Additionally, it has been used for diagnosing spontaneous bacterial peritonitis in cirrhotic patients, distinguishing exacerbated asthma in children, and differentiating between infectious and non-infectious inflammation in hemodialysis patients^18^ ^,^ ^32^ ^,^ ^33^. In 2023, Lu et al. studied the combined utility of BISAP, CRP, and NLR in assessing the severity of AP. Their study revealed that the combination of BISAP, CRP, and NLR significantly enhances the accuracy of predicting SAP, achieving a sensitivity of 86.7% and a specificity of 100%. Nevertheless, that study did not explore correlation of those markers with organ failure^34^. 

In the current cohort, the accuracy of the C-NLR score in predicting organ failure in AP was assessed. Our results showed that a C-NLR score of 2, which corresponds to patients with both high NLR and high CRP levels, was most commonly observed in cases of MSAP and SAP. Furthermore, a C-NLR score of 2 was found to be an independent predictor of organ failure, tripling the associated risk. 

The main strength of the current research is that it is the first study where it was assessed the potential utility of this score as prognosis marker of organ failure in patients with AP. Nevertheless, several potential limitations should be acknowledged. First, the retrospective design of our study inherently limits the level of evidence, and potential selection bias could not be fully excluded. Second, the study was conducted at a single center, which may restrict the generalizability of our findings. Third, we excluded patients with incomplete medical records, which could have introduced selection bias. Finally, although we accounted for key confounding factors, residual confounders may still have influenced the results. Given these limitations, we recommend conducting multicenter, prospective studies to corroborate the present findings.

In conclusion, our findings suggest that the C-NLR score could serve as a valuable complementary tool to traditional scoring systems for predicting organ failure in AP patients. Its main clinical advantage lies in its simplicity, relying on two readily available parameters, making it both practical and widely applicable.
